# COVID‐19 community assessment hubs in Ireland: A study of staff and patient perceptions of their value

**DOI:** 10.1111/hex.13603

**Published:** 2022-11-05

**Authors:** Eilish McAuliffe, Sophie Mulcahy Symmons, Ciara Conlon, Lisa Rogers, Aoife De Brún, Marese Mannion, Niamh Keane, Liam Glynn, Joseph Ryan, Diarmuid Quinlan

**Affiliations:** ^1^ IRIS Centre, School of Nursing, Midwifery & Health Systems University College Dublin Dublin Ireland; ^2^ Academic Affairs Trinity College Dublin Dublin Ireland; ^3^ Burren Medical Centre Clare Ireland; ^4^ Midwest Community Healthcare Organisation (CHO3) Limerick Ireland; ^5^ School of Medicine, University of Limerick & HRB Prmary Care Clinical Trials Network Ireland Galawy Ireland; ^6^ The Weir Clinic, Bandon Cork Ireland; ^7^ Irish College of General Practitioners Dublin Ireland

**Keywords:** community assessment hubs, coronavirus, COVID‐19, patient experience, staff experience

## Abstract

**Background:**

Critical care bed capacity per capita in Ireland is among the lowest in Europe. The COVID‐19 pandemic has put additional strain on an over‐stretched healthcare system. COVID‐19 community assessment hubs (CAHs) were established to prevent unnecessary admission to acute hospitals and to reduce infection spread.

**Objective:**

The aim of this study was to assess the effectiveness and acceptability of CAHs and identify how the service might be improved or adapted for possible future use.

**Design:**

This was a mixed methods study, incorporating co‐design with clinical stakeholders. Data collection was via an online survey and semistructured telephone interviews with staff and patients conducted between January and May 2021.

**Setting and participants:**

Thirty‐one patients completed the survey and nine were interviewed. Twenty interviews were conducted with staff.

**Results:**

The findings suggest that the CAH model was successful in providing a dedicated pathway for assessing patients with COVID‐19 symptoms, whilst mitigating the risk of infection. Patients were particularly positive about the timely, comprehensive and holistic care they received, as well as the accessibility of the clinics and the friendly attitudes of the staff. Staff welcomed the training and clinical protocols which contributed to their feelings of safety and competency in delivering care to this cohort of patients. They also highlighted the benefits of working in a multidisciplinary environment. Both staff and patients felt that the hubs could be repurposed for alternative use, including the treatment of chronic diseases.

**Discussion:**

This study describes staff and patients' experiences of these hubs. An unexpected outcome of this study is its demonstration of the true value of effective multidisciplinary working, not only for the staff who were deployed to this service but also for the patients in receipt of care in these hubs.

**Conclusion:**

This multidisciplinary patient‐centred service may provide a useful model for the delivery of other services currently delivered in hospital settings.

**Patient or Public Contribution:**

An earlier phase of this study involved interviews with COVID‐19‐positive patients on a remote monitoring programme. The data informed this phase. Several of the authors had worked in the CAHs and provided valuable input into the design of the staff and patient interviews.

## 1 INTRODUCTION

**Figure 1:**
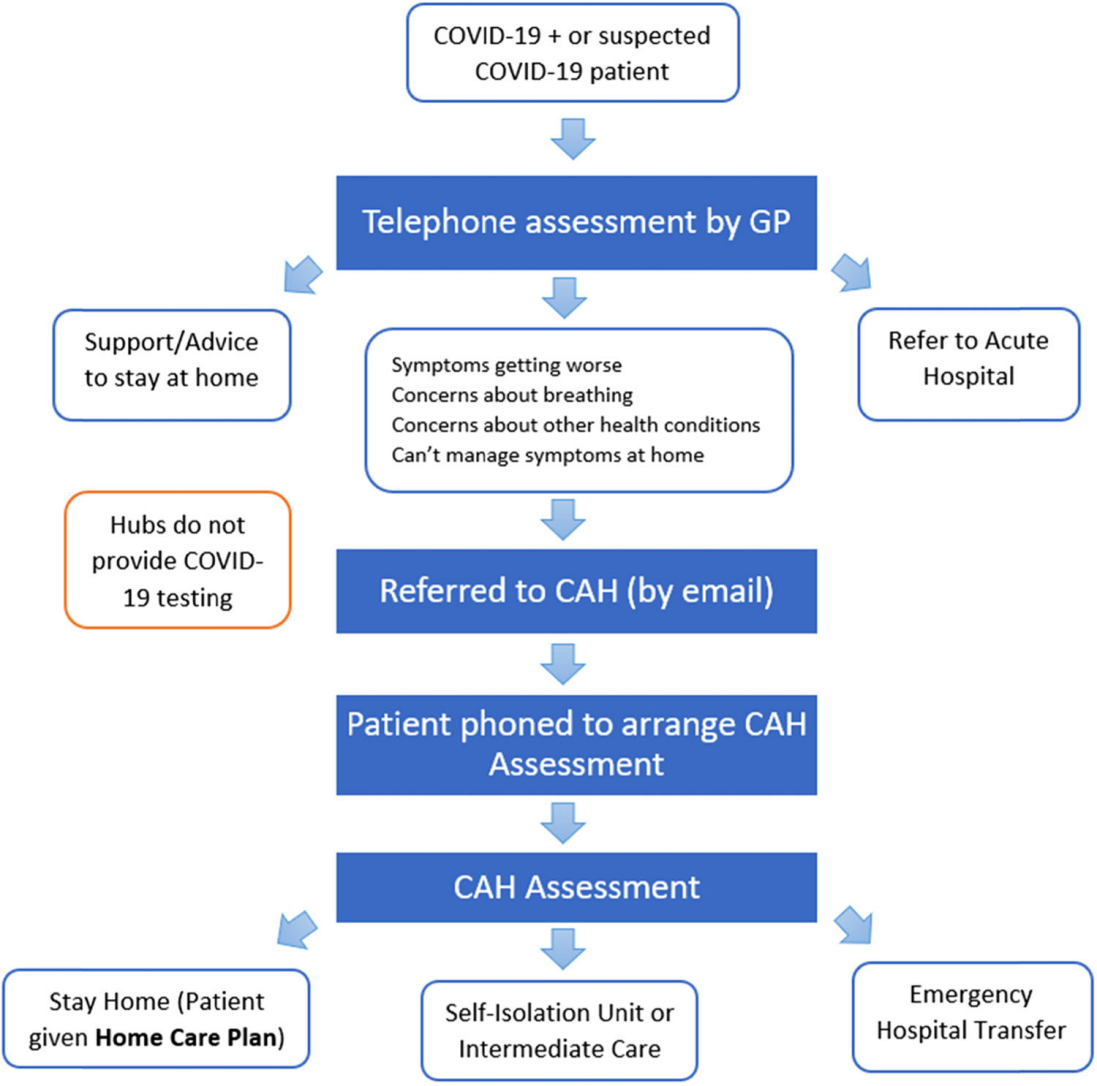
COVID‐19 community assessment hub referral pathway

The Irish healthcare system was considered poorly equipped to manage a global infectious disease pandemic. Irish hospitals operate at near full capacity on a regular basis. The low ratio of intensive care unit (ICU) beds to population size compared to other countries was of concern, given a late presentation to hospitals and subsequent rapid deterioration, resulting in more patients requiring admission to the ICU.^1^, ^2^ High levels of COVID‐19 amongst healthcare staff added to the existing staff shortages and demand on the system. In addition, Ireland's low general practitioner (GP) to population ratio^3^ resulted in primary care experiencing significant challenges managing the surges in COVID‐19‐positive or suspected positive patients. In summary, Ireland's healthcare system was not well‐equipped to manage an escalating number of people presenting with COVID‐19 symptoms, mild or severe. Thus, Ireland faced many challenges in the early stages of managing the COVID‐19 crisis. In response to these challenges, the Health Service Executive rapidly developed a novel community assessment service in April 2020, namely the COVID‐19 community assessment hubs (CAHs) to reduce infection of staff and patients in primary care services, avoid unnecessary emergency department (ED) attendance and provide timely specialized care for patients.^4^ The World Health Organization recommended that countries set up services to assess, test and treat COVID‐19 patients to combat the pandemic and the strain placed on health systems.^5^ Service models with varying levels of integration with hospitals and level of care provided, including ‘fever clinics’, have been used effectively across the globe in other infectious epidemics,^6^, ^7^, ^8^, ^9^, ^10^ but there is limited information on experiences of these services. The models vary across countries, the Irish model provides an assessment, triage and either treatment or referral. It is most similar to the primary care assessment services set up in the United Kingdom to alleviate the high demand for primary care services during the pandemic. These CAHs acted to triage patients who were COVID‐19‐positive or suspected positive. Figure 1 describes the CAH patient pathway where patients were referred by their GPs, assessed at the CAHs and then provided supportive information to isolate at home or at an isolation facility or referred to the ED if needed.^11^ CAHs consisted of a multidisciplinary team of volunteer GPs, and redeployed nurses, physiotherapists and administration staff. Approximately, 50 CAHs were established around Ireland, operating 12 h a day, 7 days a week initially, although this decreased significantly as demand reduced. The CAHs were closed in March 2021.

The COVID‐19 pandemic provoked fear and uncertainty globally. The public was fearful of the disease, falling ill, spreading the disease to their loved ones and were hesitant to attend health services.^12^ These concerns were paralleled in healthcare staff. For healthcare staff, the pandemic provoked experiences that were both positive and negative.^13^, ^14^ Some staff felt unprotected, with limited personal protective equipment (PPE) and training, yet there was an atmosphere of solidarity and learning.^13^, ^14^ It is critical to understand these experiences and learn from them to inform service reconfiguration and redeployment of staff in response to any future pandemics or similar healthcare crises.^15^

Literature emerging from the COVID‐19 pandemic has shown widespread strain on health services and staff and vulnerabilities of health systems. It highlights the need for more robust systems, developed protocols and adequate resourcing and capacity to manage in emergencies.

New services set up in emergency situations require evaluation just as services that are established over time. Despite this, they receive limited attention and therefore valuable learning may be lost. As the CAHs were a novel service, rapidly set up with little evaluation to date, it is critical to explore their effectiveness and acceptability from both the service user and staff perspective. Gaining experiential information through unveiling insight into patients' preferred care pathways and through understanding the benefits and challenges of delivering care at the CAHs is critical to this evaluation.

This paper explores the effectiveness, acceptability and experience of CAH‐delivered services from staff and patients' perspectives in two regions in Ireland. The study time period is from April 2020 through to March 2021, when the CAHs closed.

### 1.1 Aim

The aim of this study is to assess the effectiveness and acceptability of CAHs and identify how the service might be improved or adapted for possible future waves of COVID‐19 and other public health emergencies.

## 2 MATERIALS AND METHODS

### 2.1 Design

The study design chosen was a retrospective mixed methods study. The survey method was selected to gather data from as many patients as possible on acceptability, with follow‐up telephone interviews being utilized to explore patient experiences in more depth. As this was a new experience for staff, we believed it was important to allow them space to expand on their experiences through interviews rather than constraining their responses within a survey format. The method was particularly useful for exploring possible future use of the hubs. Full details of the methods are reported elsewhere.^11^ The online survey consisted of questions relating to the patient's demographic characteristics, COVID‐19 symptoms and their experience with the CAH service (including the information they received to isolate at home [four questions rated on a scale of 1 (not at all) to 5 (greatly)], access to care [rated as very easy, easy, neither easy nor difficult, difficult, very difficult], quality of care [using the Patient–Professional Interaction Questionnaire (PPIQ) scale (16 questions rated on a scale of 1–5)]).^16^ The interview guides for both patient and staff participants were designed to gain an in‐depth understanding of the acceptability and experiences they had been assessed or working at the CAHs respectively. The guides included questions pertaining to their experience including changes in clinical practice, management of patients, communication, perceptions of care and timeliness at the CAHs. The interview guide was refined during data collection based on topics of importance to the participants.^11^

### 2.2 Ethics

The study was approved by the COVID‐19 National Research Ethics Committee (Ref: 20‐NREC‐COV‐093).

### 2.3 Participants and setting

Four CAHs across two regions in Ireland were the setting for this study. There were three CAHs in region A and one in region B. All staff who worked in a CAH (114 from region A and 60 from region B) for at least a week were invited to participate in the study via email with the information leaflet and consent forms emailed through gatekeepers.

All patients who were referred to and assessed in a CAH were aged over 18 and had the capacity to consent (all 194 patients from April to June 2020 in Region A and 200 patients assessed in January 2021 in Region B) were eligible to participate in the study. Those who met these criteria and had been discharged were therefore sent, via posted letter, an invitation to participate, an information leaflet and a link to a survey through gatekeepers. The survey was hosted on Qualtrics.com, which is GDPR (general data protection regulation) compliant. Additionally, 10 patients in Region B, assessed in January 2021, who were not contacted about the survey were sent an invitation to take part in the interview only. This CAH though not included in the initial study design, indicated an interest in being included. The timeframe for completion of the project did not allow for the survey to be completed in this CAH, but patients were contacted for interviews in an attempt to increase the number of patients interviewed.

### 2.4 Data collection

Telephone interviews were conducted with all participants due to the COVID‐19 pandemic and the facilitation of social distancing guidelines and a national lockdown. Interviews took place between January and May 2021. Staff who returned the consent forms were contacted and a 20–30 min telephone interview was arranged with them at a time of their convenience. The aim was to purposively sample staff to ensure a range of views from each discipline (GP, nurse, physiotherapist, admin) were captured. Due to the low response rate, staff was convenience sampled and a representative sample of 20 staff participants from across all disciplines was obtained. Patients who completed the online survey and provided their contact details for a 20‐min follow‐up interview were contacted. Demographic data were collected from the survey. There was a low response rate for the online survey, hence convenience sampling was necessary. One researcher conducted all interviews remotely by phone, which were recorded and transcribed verbatim.

### 2.5 Analysis

Thematic analysis was conducted to explore all participants' perceptions of the CAHs and identify common themes.^18^ Coding was conducted on NVivo 12 software.^19^ Themes were drawn out inductively and reviewed and discussed with the research team. One researcher developed the initial codes from transcripts of the staff interviews. Initial themes were discussed with two other researchers and refined iteratively as more data were collected until consensus was achieved. A fourth researcher independently coded and developed themes from transcripts from patient participants, discussed with researcher 1 and integrated them with themes from the staff data. A fifth researcher independently coded a subset of transcripts to ensure the quality of the research.

Simple descriptive statistics were conducted on the 31 survey responses. Basic percentage summaries were calculated to determine the acceptability and experiences of patients attending the CAHs. The means and standard deviations (SDs) were calculated for the overall scores of the PPIQ and for the information on symptom management.

## 3 RESULTS

In total, 31 patients completed the online survey, of which, 21 indicated interest to participate in a follow‐up interview. Fourteen were subsequently contacted for interviews and nine interviews took place, five in region A and five in region B.

Twenty‐seven staff indicated an interest in participating by returning consent forms, however, 7 later declined, therefore 20 interviews with staff were conducted in total, 14 in region A and 6 in region B.

The demographic characteristics of respondents who completed the survey are displayed in Table 1. The sample was distributed across the age ranges from 30 to 60+ years with only 6% of the sample being under 30 years. The majority were female (61%), White Irish (83%) and had a university degree or above (58%). Of these patients, 27 (87%) tested positive for COVID‐19, 2 tested negative and 2 were not tested (likely due to contracting COVID‐19 early on in the pandemic when testing criteria were stricter).

**Table 1:** Characteristics of respondents who completed the survey

Age	18–29	30–39	40–49	50–59	60+
	6%	23%	26%	19%	26%
Gender	Male	Female			
	39%	61%			
Ethnicity	White Irish	White other	Asian		
	84%	3%	13%		
Education level	Lower secondary	Upper secondary	Postsecondary certificate/vocational	Degree/third level	
	10%	16%	16%	58%	

**Figure 2:**
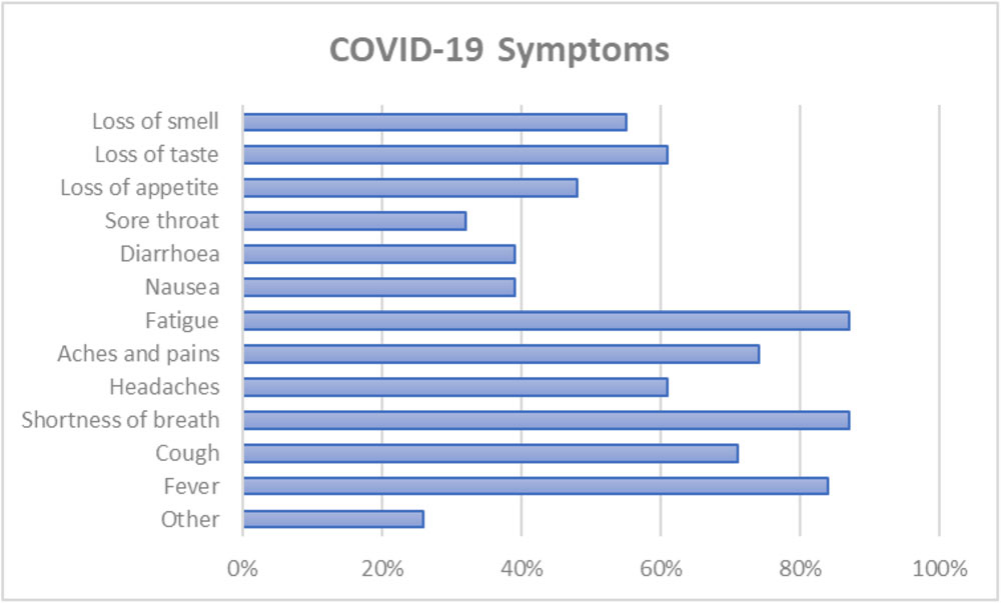
COVID‐19 symptoms experienced by survey respondents

Figure 2 displays symptoms as reported by patients. The most common symptoms patients experienced over the course of their illness were shortness of breath (87%), fatigue (87%) and fever (84%). Some patients reported still experiencing symptoms at the time of completing the survey. Patients were also asked about underlying conditions with the most commonly reported being high blood pressure (26%), asthma (23%), followed by obesity (19%), and diabetes (19%).

Table 2 provides a summary of how patients rated their experience of care. Most patients rated their experience of accessing care at the CAHs as ‘easy’ or ‘very easy’. In relation to the patient–healthcare staff interaction, patients rated the quality of care highly (the mean PPIQ rating was 72.8 [(SD = 10.7]) where the maximum score was 80. The majority of patients (71%) rated their care as better than usual, with 26% rating the service as ‘about the same’ as their usual experience of healthcare services. Most patients received information about how to manage their symptoms at home, but 29% were referred to the hospital for further investigation/treatment. Patients rated the information they received with a mean rating received 18.9 (SD = 6.9), where the maximum score was 25.

**Table 2:** Patients' experience of care received as measured through the survey (*N* = 31 respondents)

Variable	*N* = 31, *n* (%)
Ease of access	
Getting through to my GP/family doctor on the telephone	
Very easy	11 (35%)
Easy	12 (39%)
Neither easy/nor difficult	4 (13%)
Difficult	2 (6%)
Very difficult	2 (6%)
Does not apply	0
Getting an appointment at the COVID‐19 CAH	
Very easy	19 (61%)
Easy	9 (29%)
Neither easy/nor difficult	3 (10%)
Difficult	0
Very difficult	0
Does not apply	0
Getting the results of your assessment	
Very easy	18 (58%)
Easy	9 (29%)
Neither easy/nor difficult	2 (6%)
Difficult	1 (3%)
Very difficult	0
Does not apply	1 (3%)
Getting access to further care (hospital/isolation facilities)	
Very easy	10 (32%)
Easy	3 (10%)
Neither easy/nor difficult	4 (13%)
Difficult	3 (10%)
Very Difficult	3 (10%)
Does not apply	8 (26%)
Received information to isolate at home	
Yes	21 (68%)
No‐referred to hospital	9 (29%)
No	1 (3%)
	Mean (standard deviation)
Patient–Professional Interaction Questionnaire	
Total sum	72.8 (10.7)
He/she provided me with clear information	4.6 (0.87)
He/she was interested in what I feel about my current health status	4.6 (0.8)
He/she turned to me in a calm and quiet tone	4.7 (0.63)
He/she understood my emotions	4.6 (0.85)
He/she was interested in what I know about my disease/prognosis	4.5 (0.85)
He/she respected me as a person	4.9 (0.43)
He/she was interested in what I want from care	4.5 (0.85)
He/she was able to listen	4.8 (0.56)
He/she paid attention to what I was saying	4.7 (0.69)
He/she was able to put him/herself in ‘my shoes’	4.2 (0.99)
He/she gave me time to ask and to talk about the disease	4.5 (0.93)
He/she inspired confidence and security when touching me and being nearby	4.7 (0.59)
He/she asked questions that allowed me to express my view	4.4 (0.99)
He/she was interested in what I expect from care	4.4 (0.99)
He/she gave me encouragement and transmitted optimism	4.4 (1.05)
He/she offered me the opportunity to discuss and decide together the ‘things to do’	4.3 (1.25)
Information on symptom management	
Total sum	18.9 (6.9)
Increased my knowledge about COVID‐19	3.9 (1.3)
Was useful to help me manage my symptoms at home	3.6 (1.6)
Reassured me that I could manage my symptoms at home	3.6 (1.6)
Reduced my anxiety about COVID‐19	3.6 (1.6)
Reassured me that I would receive appropriate care if my symptoms worsened	4.2 (1.5)

Abbreviations: CAH, community assessment hub; GP, general practitioner.

Of the CAH staff interviewed, there were six nurses, six GPs, two physiotherapists and six admin staff. There were 15 females and 5 males, reflective of the overall gender balance in healthcare staff.^20^ Of the patients interviewed six were female and three were male. The age range most common among the patients interviewed was 30–39 (*n* = 4), while three were between 40 and 49 and the rest 50 or above. The majority of patient participants were White Irish (*n* = 6) and 8 (90%) indicated they held a degree or above. Four main themes were identified in the staff and patient interviews: (1) Efficiency of protocols and referral pathways in mitigating risk, (2) Patients' positive experience of care, (3) Positive working environment and (4) Potential for an expanded role for hubs.

### 3.1 Efficiency of protocols and referral pathways in mitigating risk

This major theme consisted of a number of subthemes namely: Mitigated exposure to risk; Specialized training; Dedicated pathways of care for COVID; and Referral systems.

#### 3.1.1 Mitigated exposure to risk

Safety was referred to many times in staff narratives as it is critical for staff when delivering healthcare, particularly in the context of a progressing pandemic. Feeling safe in accessing care was also crucial for patients, as hesitancy to access healthcare has been reported during COVID‐19, as well as in previous pandemics. Both staff and patients were satisfied with the processes related to the delivery of care, such as patient pathways and infection control protocols put in place in the hub. One staff member described being prioritized in terms of access to PPE, and many referred to the thorough clinical training around COVID and PPE they received. Efficient organization and responsive management also contributed to a sense of safety, which alleviated the worry of contracting COVID‐19:

They were very well organized. We were trained extremely well. I felt very comfortable at work and working with the team (Staff 12, Region A)

A couple of patients also described feeling reassured by the protocols in place, with one stating they felt staff were taking ‘all precautions for me and themselves’ (Patient 8).

#### 3.1.2 Specialized training

Although many staff referred to the specialized training provided on COVID‐19 and PPE and how beneficial this was in helping build their confidence in dealing with COVID patients and refreshing skills, there was variation in the standard and amount of training received across the different hubs. One participant felt a standardized training would be more appropriate to ensure staff was providing consistent standards of care across all hubs:

there was a variety of different levels of training going on. And even when we as a staff got together, and we compared the different training we had got, there was a lot of difference. Which you might think is surprising, but that was the case that was. Depending on where you got your training, you were told different things. So, that isn't a great idea. So, much more, the training needs to be made much more standardised, across everybody involved in the hubs (Staff 2, Region A)

An example of this variation ‘we did sit down in the hub one day and we had a respiratory nurse specialist speak to us which was a kind of an added‐on training if you like. It was not part of what the national guidelines was, that it was recommended training’ (Staff 7, Region A).

Others spoke of this standardized national training programme ‘we did a lot of that beforehand, that was for the month of March really, we were doing it on HSELanD (Health Services Executive training platform) constantly’ (Staff 6, Region A).

#### 3.1.3 Dedicated pathways of care for COVID

Both staff and patients felt that the service served to bridge the gap between the community and acute services, relieving the pressure on the health service. Staff felt that the hubs mitigated the risk that would have arisen had symptomatic or COVID‐positive patients been seen in the usual health facilities. Seventy‐one percent of patients reported that the dedicated CAH service was better than their usual health service. There was also a positive sentiment expressed from both patients and staff regarding the specific pathway of care the hubs provided to COVID patients, enabling staff to provide appropriate and precise care for specific symptoms:

it's very specific, the care is very specific, and the equipment is there that's needed and there's a process, you know there's no diversion away from it and it's very clear (Staff 4, Region B)

Patients were reassured by the sense that the hubs were a dedicated service, a ‘one stop shop’, where staff were trained specifically and had the expertise required to treat COVID:

…somewhere that was specialised for Covid was reassuring … to go and know that everybody there had the best knowledge that they could of this you know (Patient 6)

Patients that were advised to isolate themselves at home were provided information about how to manage their symptoms, and these patients rated the specific information they received highly (mean = 18.9, SD = 6.9).

There was widespread agreement across all participants that this specialized model of care could be repurposed for other diseases, something we will return to later in this paper.

#### 3.1.4 Referral systems

Staff felt the referral pathways with GPs and EDs were effective, and particularly expressed satisfaction with the utilization of the iNEWS (iNEWS Irish National Early Warning Score—uses a recognized scoring system to determine the degree of illness of a patient and prompts critical care intervention) score, which (because of its widespread use in EDs) improved communication with EDs and fast‐tracked patients through the triage process in EDs, reducing the wait time for patients. Patients who were referred to the ED also mentioned this as an additional benefit of attending hubs. The use of an electronic referral system was generally thought to be efficient, with clear and direct communication between services enabling good service integration, although there were some initial technological glitches and some GPs who were less proficient with technology may have found it difficult to navigate at times.

### 3.2 Patients' positive experience of care

Patients were overwhelmingly positive about their experience of receiving care in these hubs, speaking specifically about the timely access and good geographical accessibility of the hubs, as well as the high quality of care and the benefits of being attended to by several healthcare disciplines in one visit.

#### 3.2.1 Timely access and accessibility

Patients expressed high levels of satisfaction with their experience in the hubs and staff also perceived a high level of patient acceptability of care received. The majority of surveyed patients rated the ease of access to CAH services highly, with 90% of responding patients considering it easy or very easy to obtain an appointment Patients found the hubs to be very accessible, both in terms of securing appointments and physical proximity/transport to the hub. Most patients were given an appointment ‘within a few hours’ of referral, and some were provided with transportation to the hubs. Many also felt the hubs were conveniently located. Patients also appreciated how staff anticipated their arrival and dealt with them promptly, as compared to often long waiting times in EDs.

#### 3.2.2 High quality of care

Patients described pleasant interactions with staff, who were described as ‘kind’ (Patient 8) and ‘approachable’ (Patient 7) and providing a high level of reassurance. This perception of patient‐centred care was evident in the survey results, with high PPIQ ratings observed (mean = 72.8, SD = 10.7). Patients felt heard, reassured and informed, while staff observed how patients' anxiety reduced during the assessment.

I just felt that they all really listened to what I was saying and how I felt and what my experience was. (Patient 6)

There was a sense that staff was able to dedicate more time to patients and they did not feel rushed, as opposed to other services where they often feel staff are more time pressured:

There was no rush on the assessment hub staff or there didn't seem to be anyway. It wasn't to get you in and out as fast as possible, it was to get you in and have a look at you and do a thorough examination of you (Patient 5)

Overall, patients felt the staff held a high level of expertise and provided a thorough assessment of their condition, although one participant expressed uncertainty as to the seniority of the clinicians, and another was concerned about the lack of continuity of care. Staff credited the positive patient experience to the calm environment of the hub, and the low numbers of patients, in contrast to busy and overcrowded EDs, which allowed them to dedicate more time to each patient:

I think they preferred it to going to A&E where it would be a lot more crowded, and the waiting times are longer (Staff 5, Region B)

#### 3.2.3 Multidisciplinary nature of service

Both patients and staff highlighted the benefits of the multidisciplinary nature of the team providing care, which staff felt helped them provide a more holistic, patient‐centred approach to care. Staff felt patients benefited from having a multidisciplinary team that could provide comprehensive care for all of their symptoms:

having those core disciplines there, that was excellent for patients. When else would a patient see all those disciplines together unless if they were in the acute hospital? (Staff 3, Region A)

Patients echoed these sentiments, with one pointing to the availability of a physiotherapist to perform breathing exercises with them, concluding ‘I couldn't have asked for better help’ (Patient 9).

### 3.3 Hubs as a positive working environment

Both staff and patients commented on the positive nature of the hub environment, agreeing that they were well‐organized, safe spaces that fostered a high degree of collaboration and learning.

#### 3.3.1 Safe organized spaces

Staff spoke about the anxiety and fear of so many ‘unknowns’ when they were redeployed to work carefully in the hubs. Some staff described an initial discontent and apprehensiveness about the redeployment, which was challenging to deal with from a management perspective.

A lot of them were very disgruntled and unhappy and nervous about coming in. Because we were walking into the unknown (Staff 2, Region A)

However, once training was received and the hubs were operational, staff felt they provided a safe place to work. Staff expressed positive sentiments about their involvement with an innovative health service, that was shifting pressure from acute services. They referred to strong leadership and responsive management who listened to their concerns and kept the staff ‘in the loop’ (Staff 3, Region A). The overall consensus was that the hubs were organized and well managed. This was also reflected in patient narratives who commented on how ‘it was really well planned out and well organized’ (Patient 7).

#### 3.3.2 Fostering collaboration and interdisciplinary learning

It was evident that the staff felt the hubs cultivated collaborative ways of working, interdisciplinary learning and a reduction of hierarchies in the team. Despite some initial reluctance, staff described working in the hubs positively; as an interdisciplinary environment that created a shared learning culture: ‘shared learning between the different disciplines’ (Staff 3, Region A). They described gaining renewed confidence in their own skills and ability to be flexible and adaptable.

Staff felt being co‐located fostered collaboration, particularly those who typically worked independently. Most described ‘a strong sense of collegiality’ (Staff 2, Region A) and ‘team spirit’ (Staff 8, Region A), assisting colleagues with PPE. Participants felt this facilitated them in building rapport and establishing new working relationships which their role would not usually facilitate. Some participants felt the hub environment lent itself to reduced hierarchies among the disciplines, and a sense of shared decision‐making with more information sharing and consultation between different disciplines:

I was included in the conversation and probably that would never happen in my previous role, but it was yeah I found it inclusive […] we were all linked together, there was that sort of camaraderie feeling about it and everyone supported each other because it was the unknown you know (Staff 10, Region A)

Participants also felt the experience of working together in the hub has facilitated the building of both professional and personal relationships with other healthcare workers in the community, which has proved to be beneficial in community patient management since the hubs closed, for example when referring patients to other services:

Sometimes in the community, doctors, and nurses… you know it's very cut and dry whereas now, you've that relationship with them (Staff 6, Region A)

#### 3.3.3 Potential for an expanded role for hubs

Participants (both patients and staff) were specifically asked if the hub model might be useful for use in the future and for purposes other than COVID‐19 assessment. Several commented on the demand and supply imbalance and some frustration was expressed at what was perceived as an underutilization of the hubs. Despite this, many staff and patients felt the hubs could be adapted for use in the management of chronic diseases, to reduce unnecessary reliance on EDs and for other situations where infection control might be paramount. Staff emphasized the need for additional resourcing of the hubs to render them suitable for the expansion of their use into these other areas.

#### 3.3.4 Underutilization of hubs

The peaks and dips in COVID‐19 infection rates meant predicting the numbers of patients that might attend the hubs and resourcing the hubs accordingly was a difficult task for management. One positive result from this was that low demand provided a better care delivery experience for patients, with staff having more time to attend to individual patient needs.

the level of care was excellent, the expertise was spot on, they were courteous, there wasn't a feeling of being processed, it was a matter of people were listening and they were interested and they were, it wasn't a matter of here's your script and off you go, it was a totally different attitude altogether (Patient 9)

However, some staff expressed frustration at the low volume of patients, particularly staff who had been redeployed from services that had increasing waiting lists. Some thought the hubs were underutilized and sometimes inappropriately used by GPs for several reasons including GPs not having enough awareness about the hubs and their role, the financial loss GPs would incur by referring a patient, or a desire to treat their patients on their own practice rather than refer to the hubs. Staff raised questions about the financial viability of the hubs due to low demand:

when you divide that [cost of the service] by the number of patients seen, fairly expensive assessments (Staff 5, Region B)

#### 3.3.5 Providing more holistic care for chronic conditions

Both the patient group and the hub staff remarked on the opportunity to provide more holistic care with several disciplines in one site and how this could be a good model for future services.

and a small team you know of you know GP, physio, and specialist nurses you know you could certainly handle a lot of stuff. You know and then keep it in the community (Staff 4, Region A)

Several staff mentioned diabetes as a condition that could benefit from this holistic multidisciplinary care model.

diabetic patients. If they were able to come and have their outpatient care, like if they were attached to a particular hub… I mean if they had to come for say wound care, dressings, you know skin care, you know you'd obviously have tissue viability Nurses, or you know Public Health Nurses there who might be able to look at that, and then if there's issues around general health and physical fitness and, you know a lot of diabetic patients, for example, end up with particular things like neuropathy, frozen shoulders—things that Physiotherapists might help with—and the same an Occupational Therapist might have a role in terms of helping them with particular equipment or activities that they need to do, and then the Doctor would look at managing of their blood sugars and all of that, and I suppose it would be just nice to have a whole team collaborative approach, rather than having to go to the acute hospital. (Staff 8, Region A)

The management of other chronic respiratory or cardiac conditions through community hubs was also mentioned by several staff.

people with chest problems could go there, especially for the physios and that you know and even just dedicated clinics really, that's what it would be very good for, even leg ulcer clinics, just different clinics you could bring people back to one place with an IT system that works well (Staff 6, Region A)

I think there's definitely a big gap in the community for not just respiratory, and I definitely think you know, a lot of the patients that are in A&E don't need to be there. They need to be assessed in somewhere like a Hub (Staff 5, Region A)

#### 3.3.6 Reducing utilization of EDs

This perception that there are many patients who attend EDs that do not need to be there, was echoed in the patients' comments. They were particularly positive about the time saved:

the nature of the expertise that was there locally without the endless ED waiting time because that's probably where my GP would have sent me next (Patient 9)

Patients were also concerned about not utilizing the scarce resources in hospitals when they could be effectively treated elsewhere and felt that the hubs facilitated this.

I definitely think those units are an amazing idea because if the GPs can't see you, you are very sick like that, you don't want to be going to the hospital where there are people sicker than you are, do you know what I mean, obviously I didn't want to be taking up a bed that I didn't need. (Patient 4)

There was therefore strong consensus between patients and staff that these hubs could play a vital role in reducing the number of patients that may be attending EDs when their condition could be more efficiently treated in a community setting. Some staff did, however, draw comparisons to the Medical Assessment Units in acute hospitals and were concerned that utilizing the hubs to reduce the burden on EDs may just result in a duplication of the work that these established units are currently undertaking.

#### 3.3.7 Infection control and expert knowledge

Staff repeatedly referred to the quality of infection control measures and the training in the management of COVID that they received when deployed to work in the hubs. As already highlighted, many staff commented on a feeling of being protected and being in a safe working environment. Patients were also reassured by the expert knowledge of the staff and the measures that were being taken to ensure the risk of infection was minimized.

it was very, very reassuring to go and know that everybody there had the best knowledge that they could of this you know. (Patient 6)

Given the high‐quality infection control and expertise of staff, it is not surprising that many of those interviewed were in agreement that this model of service delivery should be replicated for any future pandemic or outbreak of infectious disease.

#### 3.3.8 Further resourcing of hubs

Many participants commented on the absence of diagnostic tools, such as X‐ray machines and blood testing requests, and felt that without these the hubs were not being used to the maximum potential for COVID patients, and the absence of such tools would limit their utility for other conditions.

It's called the respiratory hub and that should have all the facilities for what you are being assessed for (Patient 1)

Patients thought these resources would have streamlined their care experience and prevented them from being referred to EDs. Some staff also felt limited in what they could do for the patients.

Many participants felt the hub model, if better resourced, could be applied to other conditions to create more specialized holistic and patient‐centred care using a multidisciplinary approach.

some of them just might need bloods and chest X‐ray to see you know and check their oxygenation and see are they safe at home you know, can they be managed at home and could the community intervention team then give IV‐antibiotics at home you know, save a bed you know (Staff 4, Region B)

There were also suggestions about staffing resources that would be required should the hubs be used as specialized clinics:

GPs who have a special interest in a certain thing could be brought in to run a clinic or day hospital you know … and you could have a consultant in there maybe for the afternoon and you could have lots of day cases to do you know and—so I think that model you know is a good model (Staff 10, Region A)

One staff member felt that there was a specific gap in paediatric care that CAHs could usefully fill.

We have the medical assessment unit and the surgical assessment unit and the local injury clinic for adults, but something, yeah, like a paediatric assessment unit would be helpful and run in probably a similar way … Yeah. Well not necessarily a GP, I suppose, but maybe a paediatric doctor or a GP that specialises in paediatrics (Staff 11, Region A)

## 4 DISCUSSION

This study adds to the limited evidence on the acceptability of COVID‐19 primary care services from both the staff and patient perspectives. Only one study has assessed the CAHs in Ireland to date,^17^ which found high compliance with infection control procedures at the hubs, as well as reported positive experiences of staff working at these hubs, corresponding to the findings of this study. In the United Kingdom, where similar primary assessment centres were established, staff reported improved knowledge of COVID‐19 and confidence in assessing patients.^10^

The findings of this study suggest that the CAH model was successful in providing a dedicated pathway for the assessment of patients with COVID symptoms and in mitigating the risk of infection associated with being assessed within the established health facilities, that is, general practice clinics or EDs. Patients and staff articulated the many positive aspects of the model, with particular emphasis on feelings of safety and being afforded the time to provide/receive good quality care. The shared sense of safety highlights the success of the clinical protocols in overcoming the initial fear associated with COVID‐19. The overwhelmingly positive experiences of staff who participated in this study do not correspond with previous research which found a negative psychological impact of the COVID‐19 pandemic on frontline workers and healthcare professionals.^13^, ^21^ This may be due to specialized training, efficient protocols in place and a sense of physical safety maintained by the teams' diligence in complying with all recommended infection control measures.

To make sense of the success of the CAH model, it is useful to draw on integrated theories of urgent care. Turnbull et al.^22^ in their conceptual model of urgent care sense‐making and care seeking, outline three distinct types of ‘work’ or thinking processes that patients typically navigate before accessing urgent care: (i) ‘illness work’—When people make sense of illness by interpreting the severity of symptoms, assessing risks and making decisions about accessing services; (ii) ‘moral work’—Work undertaken to present as an appropriate, legitimate or responsible user of healthcare—‘a credible patient’; and (iii) ‘Navigation work’—Identifying and making sense of the range of services on offer and how to access healthcare services. Illness work is, to a large extent, influenced by the level of health literacy the patient has and their knowledge about patterns of symptomatology and indicators of risk. During the COVID‐19 pandemic in Ireland, extensive efforts were undertaken through public campaigns to ensure that the general population was fully informed of the signs and symptoms of COVID‐19 and clear instructions were provided on the level of severity at which one should seek healthcare and from where one such seek such care. It could therefore be argued that patients had to perform less illness, moral and navigation work to access the CAHs than would have been the case to access EDs. Thus resulting in more efficient utilization of the CAH services.

This efficiency is evident in patients' positive comments about the comprehensive and holistic care they received in a timely manner, as well as the easy accessibility of the clinics and the friendly attitudes of staff. For many, the experience contrasted sharply with the busy, overcrowded nature of EDs (often serving as a point of access for patients that could be more effectively treated in other settings), where staff has little time to spend with individual patients due to workload pressures, and where patients may endure long waiting times in less than ideal conditions. Whilst there is a clear association between staff stress, burnout and poor quality of care measured through patient safety errors,^23^ the impact of relieving stress on staff and the potential impact on patient care is not as well researched. What this study demonstrates is that when staff is working in a well‐managed environment, where they feel adequately prepared for their role and are afforded the time to deliver care, the result is patients who feel listened to and respected, and whose experience is one of receiving high quality, holistic care. In addition, the findings make a strong argument for providing more information to the general public on what, when, and how to access ED services.

The multidisciplinary nature of the hubs also elicited positive sentiments towards the working environment. The pandemic has shown that, despite the pressure, there has been great resilience and solidarity in healthcare staff^13^ working in new environments with new colleagues. A surprisingly positive finding from this study was how novel and well‐received multidisciplinary working was by hub staff. The hierarchical culture that is typical in many healthcare settings, seems to have been suspended in these settings with many staff noting the opportunity to contribute to problem‐solving and decision‐making in a manner that would not be typical in their usual roles. A study of healthcare staff narratives of implementing change during the COVID‐19 pandemic found that the focus was on completing the task, and the position in the hierarchy was less relevant as all team members relied on each other for clinical and emotional support.^15^ This absence of hierarchy and camaraderie in the teams led to staff feeling supported, and encouraged to work and learn together as well as building professional relationships along the way. Another study where healthcare staff was surveyed on their experiences during the COVID‐19 pandemic, reported that the interprofessional collaboration enabled an atmosphere of psychological safety and creativity, where ideas and innovations were actively sought and developed collectively.^24^ This sense of psychological safety is also evident in the CAHs as the staff was able to express opinions and there was a levelling of traditional hierarchies. These unsolicited positive comments on collaboration, multidisciplinary working and levelling of hierarchies do, however, raise the question as to why this way of working is not by now the norm in healthcare, particularly given the strong emphasis on integration of care and primary care teams and networks in Ireland's health strategy.^25^

Despite the positive outlook of the CAHs, several issues were brought to light, primarily around matching resources with the demand. Nearly all staff highlighted that the CAHs were over‐resourced, particularly at the beginning of the pandemic where COVID‐19 cases were lower than expected. Staff numbers were then reduced but the staff was sometimes left with little work due to low patient throughput and the limited role CAHs played in COVID patient care, that is, they had limited ability to conduct diagnostic work other than assessing COVID symptoms. For further development of the CAHs to be a viable option, they would need to be given more powers to do more detailed assessments or provide care or treat patients. The staff view generally was that their skills and time could have been better utilized. Greater awareness of the existence and function of the CAHs amongst GPs would also assist in expanding their use. The scope of practice and the cost‐effectiveness of this model of service is something that requires further research to understand if it does have a place in the healthcare system.

Staff and patients were in agreement that this model could be used to treat patients with minor conditions or chronic illnesses who may need to be seen by an array of professionals or those who need more specialized care than a GP practice can give, but who are not so unwell as to require emergency care. The complexity of COVID‐19 has highlighted that multidisciplinary teams may be required to provide care to patients with long‐COVID symptoms. Participants identified the lack of follow‐up care for these patients. Statistics from the UK Office for National Statistics (ONS) suggest that 13.7% continued to experience symptoms for at least 12 weeks.^26^ However, a retrospective study of 273,618 patients with COVID‐19 found that in the 6 months after a COVID‐19 diagnosis, 57% had at least one feature of long‐COVID recorded.^25^ In the 90‐ to 180‐day ‘long’ phase postdiagnosis, over 36% had a long‐COVID feature recorded. Additionally, the study found that the incidence of ‘any’ long‐COVID feature varied from 46% in the 10‐ to 21‐year age group, to 61% in the over 65s.^27^ The CAHs may therefore provide the basis for a model for long‐COVID care. One such integrated multidisciplinary model of care for post‐COVID pneumonia hospitalized patients outlines the patient's physical and psychological support needs.^28^ Another study co‐designed a potential long‐COVID pathway with healthcare professionals and long‐COVID patients demonstrating that this patient group's complex care needs require a holistic ‘one‐stop‐shop’ with multiple disciplines' expertise.^29^ There is a need for continuation of care for these patients, particularly those who were not hospitalized, to ensure they do not fall through the service gaps.

### 4.1 Limitations

There were a number of limitations to this study. Engaging with busy healthcare professionals throughout peaks and dips in the pandemic proved difficult and flexible remote meeting arrangements and sustained open dialogue across sites were used to facilitate collaboration and reduce the burden on the team. This inevitably caused delays in the data collection. Our ethics permissions required that recruitment of patient participants to the study needed to be conducted through the healthcare facilities to avoid sharing patient contact information with the research team. Communicating with patients about the study placed an additional burden on staff and because of this in Region B, the decision was taken to reduce the sample size to 100 patients. An additional limitation was that patients were contacted via posted letters, as email addresses were not recorded for all patients. These limitations may have negatively impacted the response rate. The research team was not allowed to send reminders because of the aforementioned ethics stipulation regarding patient contact. An additional constraint on the research team's ability to expand the sample size was the short time frame for the study, initially 7 months but subsequently extended by 2 months. The resultant small sample size for the survey results is a significant limitation of the study. For some patients, there was several months time gap between attending the clinic and completing the study, so responses may be influenced by potential recall bias. It must also be noted that this study was conducted on a sample of CAHs and does not represent the national picture. Most of these CAHs were relatively quiet throughout the pandemic, other CAHs may have different experiences based on demand and variations in training as this was not standardized nationally.

## 5 CONCLUSION

This study provides an understanding of the challenges of delivering care to COVID‐19 patients, mitigating the risk of cross‐infection whilst providing a service without access to diagnostic capabilities. It demonstrates what is important to patients who contract an infectious disease about which they have limited knowledge. It also highlights the importance for staff of working in a safe environment and having the knowledge to deliver quality care to their patient cohort. An unexpected outcome of this study is its demonstration of the true value of effective multidisciplinary working, not only for the staff who were deployed to this service but also for the patients in receipt of care in these hubs. This multidisciplinary patient‐centred service provides evidence of the benefits of such models of care, and important learnings for their implementation. This has relevance to proposed healthcare programmes pertaining to long‐covid, chronic disease and integrated care in a community setting. However, further research is needed to assess the cost‐effectiveness of this model of service.

## AUTHOR CONTRIBUTIONS

Eilish McAuliffe contributed to the conceptualization of study, funding acquisition, data collection, data analysis and drafting and reviewing the paper. Sophie Mulcahy Symmons collected the data and contributed to the data analysis and drafting of the paper. Lisa Rogers contributed to the data analysis. Ciara Conlon contributed to the data analysis and reviewed the paper. Aoife De Brún contributed to funding acquisition, reviewed the paper. Marese Mannion assisted with recruitment of participants and reviewed the paper. Niamh Keane assisted with recruitment of participants and reviewed the paper. Liam Glynn contributed to the study design, funding acquisition and reviewed the paper. Joseph Ryan assisted with recruitment of participants and reviewed the paper. Diarmuid Quinlan contributed to the data collection and reviewed the paper.

## CONFLICT OF INTEREST

The authors declare no conflict of interest.

## Data Availability

Participant consent was obtained to utilize their data for this study's purpose only. The data therefore cannot be made available to other researchers for use.
